# Association of education level with diabetes prevalence in Latin American cities and its modification by city social environment

**DOI:** 10.1136/jech-2020-216116

**Published:** 2021-02-04

**Authors:** Ariela Braverman-Bronstein, Philipp Hessel, Catalina González-Uribe, Maria F Kroker, Francisco Diez-Canseco, Brent Langellier, Diego I Lucumi, Lorena Rodríguez Osiac, Andrés Trotta, Ana V Diez Roux

**Affiliations:** 1 Urban Health Collaborative, Drexel University Dornsife School of Public Health, Philadelphia, PA, USA; 2 School of Government, Universidad de los Andes, Bogota, Colombia; 3 School of Medicine, University of Los Andes, Bogota, Colombia; 4 INCAP Research Center for the Prevention of Chronic Diseases, Institute of Nutrition of Central America and Panama, Guatemala City, Guatemala; 5 CRONICAS, Center of Excellence in Chronic Diseases, University Peruana Cayetano Heredia, Lima, Peru; 6 School of Medicine, University of Chile, Santiago, Chile; 7 Institute of Collective Health, National University of Lanus, Buenos Aires, Argentina

## Abstract

**Background:**

Diabetes prevalence continues to increase in urban areas of low-income and middle-income countries (LMIC). Evidence from high-income countries suggests an inverse association between educational attainment and diabetes, but research in LMIC is limited. We investigated educational differences in diabetes prevalence across 232 Latin American (LA) cities, and the extent to which these inequities vary across countries/cities and are modified by city socioeconomic factors.

**Methods:**

Using harmonised health survey and census data for 110 498 city dwellers from eight LA countries, we estimated the association between education and diabetes. We considered effect modification by city Social Environment Index (SEI) as a proxy for city-level development using multilevel models, considering heterogeneity by sex and country.

**Results:**

In women, there was an inverse dose–response relationship between education and diabetes (OR: 0.80 per level increase in education, 95% CI 0.75 to 0.85), consistent across countries and not modified by SEI. In men, Argentina, Brazil, Colombia, Chile and Mexico showed an inverse association (pooled OR: 0.92; 95% CI 0.86 to 0.99). Peru, Panama and El Salvador showed a positive relationship (pooled OR 1.24; 95% CI 1.04 to 1.49). For men, these associations were further modified by city-SEI: in countries with an inverse association, it became stronger as city-SEI increased. In countries where the association was positive, it became weaker as city-SEI increased.

**Conclusion:**

Social inequities in diabetes inequalities increase as cities develop. To achieve non-communicable disease-related sustainable development goals in LMIC, there is an urgent need to develop policies aimed at reducing these educational inequities.

## Introduction

According to the WHO, diabetes prevalence has doubled over the past 30 years reaching 8.4% in 2014, with 75% of the cases occurring in low-income and middle-income countries (LMIC).^1^ Although diabetes risk factors such as body mass index appear to be increasing in both rural and urban areas,^2^ in many LMIC urban living may be especially conducive to diabetes through living and working environments; promoting sedentary work, processed foods consumption, and automobile transportation, all of which may impact risk factors for diabetes.^3–5^

In 2012, there were 62 million people with diabetes in Latin America and the Caribbean (LAC), this number is predicted to increase to 109 million by 2040.^6^ The LAC region is highly urbanised, with a substantial proportion of persons with diabetes residing in cities.^7^ Evidence from high-income countries (HIC) suggests that diabetes is strongly linked to socioeconomic disadvantage^8^ but studies of the social patterning of diabetes in large and growing cities of LMIC remain scant.^9^ Some evidence suggests that social gradients in diabetes are larger in more urbanised LMIC or in more urbanised regions within LMIC.^10–12^

Several factors may contribute to diabetes inequities in urban areas. Urban living (and changes in income and work associated with it) has been linked to more processed foods consumption, and higher prevalence of drinking, smoking and sedentary lifestyles.^9,13^ This increase initially occurs among the wealthiest populations but later transitions into a disproportionate increase in disadvantaged populations.^14–17^

Despite the importance of diabetes in urban areas and the documented presence of stronger diabetes inequities in more urbanised areas,^9–11^ no prior research has examined how specific features of urban areas affect the social patterning of diabetes in cities. Similarly, to what has been observed for countries, cities that have higher incomes, better infrastructure (including housing), and higher proportion of people with higher education levels may also paradoxically exhibit greater educational inequalities in chronic disease risk,^18,19^ because beneficial resources and environments may be unequally distributed. Understanding and documenting educational inequities in diabetes in LMIC and its variations across different urban environments is necessary to develop effective interventions to reduce the prevalence of diabetes across rapidly growing cities of LMIC.

Using a harmonised dataset of individual and city-level data for 232 cities in eight Latin American countries, we investigated educational differences in diabetes prevalence by gender, whether these inequities vary across countries and cities, and the extent to which they are modified by city social environment.

## Methods

Data are from the Salud Urbana en America Latina Project project, which has compiled and harmonised health, social and built-environment data from 371 large cities (population ≥100 000 in 2010) from 11 countries.^20^ These analyses are based on a 232 cities subset from 8 countries with available survey data on diabetes: Argentina, Brazil, Chile, Colombia, El Salvador, Mexico, Peru and Panama. Surveys were designed for chronic disease surveillance using random probabilistic household sampling. Sample sizes for countries ranged from 1333 to 30 425 participants. Survey details are provided in online supplemental table 1.

The main outcome was self-reported diabetes in adults 25 years or older. Survey participants were classified as having diabetes if they reported being given a diabetes diagnosis by a healthcare provider. Female participants who reported a diagnosis of gestational diabetes were not considered to have diabetes, except in the case of Argentina and Panama because information on pregnancy status during the time of diagnosis was unavailable.

The two key exposures investigated were city Social Environment Index (SEI) and individual-level education, a proxy of individual-level SES.

The SEI combines four variables representing various aspects of the city social environment: water access (% households with access to piped water); sanitation (% households with access to a municipal sewage network); overcrowding (% households with >3 people per room); and primary education completion (% population ≥25 years with at least completed primary education). This measure was created by summing the standardised Z-scores of the four variables (overcrowding was reverse coded). The SEI is used as a global metric of city social environment since it captures various aspects of city-living conditions. It has been shown to be related to differences in life expectancy across cities in the sample.^18^ For descriptive analyses, the entire sample was divided into SEI tertiles (low, medium, high).

Individual-level education was obtained from survey reports. Data were harmonised using the Integrated Public Use Microdata Series^21^ harmonisation definitions and categorised into the following categories: (1) less than primary: individuals with no education or less than completed primary education; (2) primary: individuals who completed primary education, but with incomplete secondary education; (3) secondary: individuals with complete secondary education, complete non-university postsecondary education (eg, technical school), or with incomplete university education; (4) university or higher: individuals who completed a university degree or with complete/incomplete graduate studies.

Because descriptive analyses suggested a linear pattern in the association between education categories and diabetes, education was examined as a continuous variable ranging from 0 to 3 reflecting increasing education categories. Age (range 25–97 years) and sex were included as covariates.

### Statistical analysis

Descriptive statistics for all predictors are presented by city-SEI tertiles and diabetes status. We estimated age-adjusted proportions by education categories and gender separately for each country using Poisson regression models. We inspected patterns (ie, whether they were approximately graded or exhibited thresholds) as well as major differences across countries in the nature of the association (eg, inverse, none, positive).

We estimated the association of city-SEI and individual-level education with diabetes using two-level mixed logistic regression models stratified by sex with individuals nested within cities, adjusting for country fixed effects. We included a random intercept for each city and a random slope for education to allow education gradients to differ between cities. Model 1 included the two key predictors city-SEI and individual-level education, as well as age. Model 2 included country fixed effects. In model 3, we included interactions between education and country fixed effects to determine if there were differences in education gradients by country. If the interaction was statistically significant, we further examined heterogeneity by grouping countries into two groups based on educational patterns observed in descriptive analyses (group 1, displaying an inverse crude association: Argentina, Brazil, Chile, Colombia, Mexico; group 2, displaying a positive crude association: Peru, Panama, El Salvador) and including an appropriate interaction term.

To test our hypothesis that city-SEI modified educational differences, we fitted model 4 which added an interaction term between city-SEI and individual-level education (both as continuous variables). Using this model, we estimated the effect of a one-level increase in education for people living in cities at the 10th, 50th and at the 90th percentiles of city-SEI. We plotted the predicted probabilities resulting from the model according to education level by city-SEI.

All analyses were stratified by sex considering previous evidence suggesting that the effects of social environment and education on non-communicable diseases (NCDs) vary by sex.^22^ For the models, city-SEI was standardised to a mean of 0 and a SD of 1. We specified a level of significance of 0.05 and all analysis was conducted using SAS 9.4 and R V3.6.1 software.

## Results

Our sample included 110 498 survey participants in 232 cities from eight Latin American countries. There was a median of 300 individuals per city (IQR 120–600). Table 1 shows city and individual-level characteristics according to city-SEI. The full sample had a median age of 43 years and 59% were women. The highest percentage of survey participants lived in Brazil and Mexico (27.5% and 21.2%, respectively), while El Salvador and Chile had the lowest (1.2% and 2.2%, respectively). Age and gender did not differ across SEI tertiles. The percentage of people with diabetes was 7%, 9% and 8%, respectively, at the lowest, middle and highest city-SEI tertile. Higher SEI cities tended to have a larger proportion of inhabitants within higher educational categories. Persons with diabetes lived in cities with a slightly higher SEI were older and more concentrated in the lower education categories than those without diabetes (online supplemental table 2).

**Figure 1:**
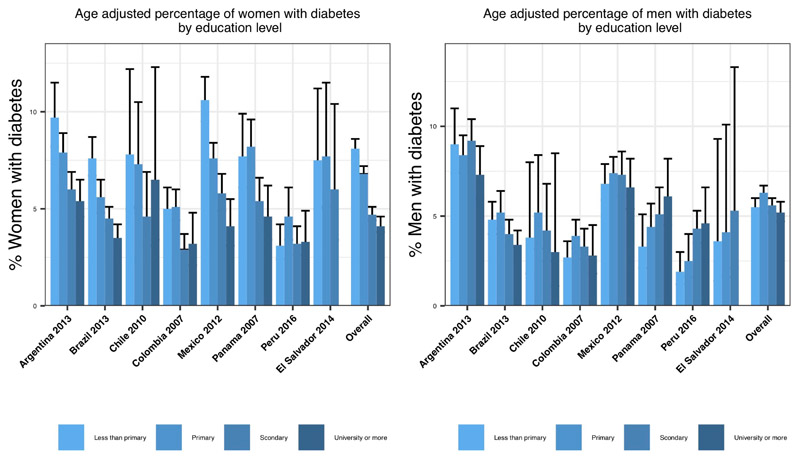
Age-adjusted percentage of people with diabetes by country and gender according to their individual education level. Age was categorised into three groups: 25–39, 40–64, and 65 and older. These categories were further included in Poisson regression models to estimate diabetes prevalence by education levels.

Figure 1 shows the age-adjusted proportion of respondents with diabetes by education level by sex. In women, there was a clear inverse association between diabetes and education for all countries except Peru; additionally, in Argentina, Brazil and Mexico there was a dose–response pattern. In men, there was an inverse association in Argentina, Brazil and Chile without a consistent dose–response relationship, but no clear pattern was observed in Mexico and Colombia, although a slightly higher prevalence of diabetes was observed in the lower educational categories. In men living in Peru, Panama and El Salvador, there was a positive association with a dose-response pattern: the higher the education level, the higher the diabetes prevalence.

Given the patterns observed in figure 1, we tested for multiplicative interactions between education and country. The global test for education-country interaction was statistically significant in men (p=0.005) but not in women (p=0.4). We also tested for heterogeneity across sets of countries with different patterns based on visual inspection of figure 1. In women, we found no evidence that associations differed significantly in Peru (p for interaction=0.8) or Chile, Colombia, Panama and El Salvador (p=0.2) compared with Argentina, Brazil and Mexico. Consequently, we estimated associations of education with diabetes pooling across all countries but adjusting for country fixed effects. In men, we found that associations differed significantly in Peru, El Salvador and Panama compared with the other countries (p for interaction=0.002). Hence, we provide two estimates of education associations: one for Colombia, Mexico, Argentina, Brazil and Chile, and another for Peru, El Salvador and Panama.

Table 2 shows the ORs of diabetes associated with individual-level education and city-SEI. In women, higher individual-level education was strongly and inversely associated with lower odds of diabetes, after adjusting for country fixed effects (OR 0.80; 95% CI 0.75 to 0.85). No associations were observed for city-SEI. The random effects for the intercept and education slope remained significant even after adjusting for country, providing evidence of residual variation in diabetes and the effect of education across cities.

In men, after adjusting for country fixed effects higher individual-level education was associated with lower odds of diabetes in Argentina, Brazil, Chile, Colombia and Mexico (0R 0.92; 95% CI 0.86 to 0.98). For Peru, Panama and El Salvador, the association was reversed, the odds of diabetes increased by 24% per one-unit higher education level (OR 1.24; 95% CI 1.04 to 1.49), after adjusting for city-SEI, age and country fixed effects. City-SEI was not associated with diabetes. The random intercept remained significant suggesting of residual variability in diabetes between cities. The variance of the random slope for education was no longer statistically significant when we introduced the interaction between education and country.

Table 3 shows associations of individual-level education with diabetes for cities with different levels of SEI. In women, there was no evidence of SEI-education interaction (p = 0.834) and the odds of diabetes were reduced by 20% for each one-unit increase in education level across the different SEI levels. The random slope for education remained statistically significant suggesting residual variation in the effect of education across cities.

**Figure 2:**
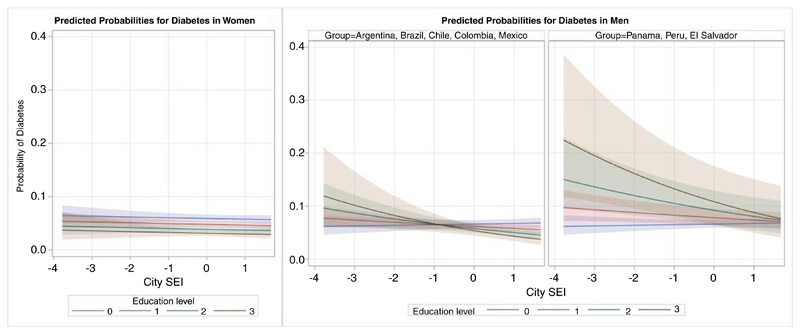
Predicted probabilities of diabetes based on the multilevel logistic regression models to assess the effect modification of education level by city-Social Environment Index (SEI). Education level: 0=less than primary; 1=primary; 2=secondary; 3=university or more. City-SEI values are based on the standardised variable used in the model so the range of values differs from the on presented in table 1.

In men, there was a statistically significant interaction (p = 0.019) between education and city-SEI. We present the OR of diabetes associated with education separately for the two sets of countries given qualitative differences in the directions of the associations between education and diabetes. Men in Argentina, Brazil, Chile, Colombia and Mexico had an inverse association of education with diabetes at higher levels of SEI but it reversed and became non-significant at lower levels (OR (95% CI) 1.03 (0.92 to 1.16); 0.90 (0.85 to 0.97) and 0.85 (0.78 to 0.93) for the 10th, 50th and 90th percentiles of SEI, respectively). For men in Peru, Panama and El Salvador, the positive association of education with diabetes weakened and became non-significant as city-SEI increased (OR (95% CI): 1.33 (1.10 to 1.61); 1.17 (0.97 to 1.41) and 1.09 (0.89 to 1.35) for the 10th, 50th and 90th percentiles, respectively). The variance of the random slope for education was reduced compared with the model without the SEI-education interaction and was not statistically significant. Figure 2 depicts these patterns graphically for predicted probabilities derived from the model with interactions.

## Discussion

We used survey data for 232 large cities in eight Latin American countries to characterise educational differences in diabetes prevalence and examine the extent to which these inequities varied across countries and cities. We also examined whether city social environment modified the relationship between education and diabetes. In women, we found a clear educational gradient; women with higher education levels had a lower proportion of diabetes, with a dose–response pattern for all countries (except possibly Peru). Furthermore, this association was not modified by city-SEI. In men, the association of education with diabetes varied across countries; in some countries, there was an inverse association while in other countries there was no association or the opposite association (higher proportion of diabetes in men with higher education). Moreover, this association was modified by city-SEI such that an inverse association emerged (or a positive association weakened) as the city-SEI improved.

Evidence from HIC consistently shows a higher prevalence of diabetes in people with lower education levels compared with higher education and this association is consistently stronger in women.^22–24^ Limited data from LMIC suggest heterogeneity in social gradients.^12,25–27^ We found that among Latin American women in urban areas, the association of education with diabetes follows the same pattern found in HIC.^22^ For men, the pattern is not constant across countries. In the case of Peru, El Salvador and Panama, we found a higher prevalence of diabetes among men with higher education.

Other research from HIC and some Latin American countries has documented stronger inverse educational or socioeconomic gradients in obesity, diabetes, and NCDs in women but weaker or opposite gradients in men.^12,16,22,23,25,27–29^ Education may be more beneficial to women because they may lack other resources such as earnings, power and authority.^30^ Social norms regarding weight, physical activity and diet (all risk factors for diabetes) maybe more strongly patterned by education in women than in men resulting in differential associations of diabetes with education by sex.^28^

In our study, country modified educational gradients in men but not in women. The countries in which we observed that higher education was associated with more diabetes in men— including Peru and El Salvador—are the countries with the lowest income per capita in our sample.^31^ These findings are consistent with prior work showing that country economic development modifies social gradients in chronic disease risk.^4,25^ In the early stages of the nutritional transition, men with higher socioeconomic status tend to be less physically active and consume more processed foods with high content of fat and salt, compared with men in lower socioeconomic status. As countries develop, this pattern reverses and those with higher income and education have healthier diets and behaviours.^9,17,32,33^ Why this modification of educational gradients by country was more evident in men than in women is a question that deserves additional research.

Our sample includes 232 cities from eight Latin American countries, with large heterogeneity in social environments. Thus, we expected our results to vary between cities. No effect modification was observed in women. In men, we found that city-SEI modified the association of education with diabetes. In countries with an inverse association, it became significant and was stronger as city-SEI increased. In countries where the association was positive, it became weaker as city-SEI increased. These results mimic the country-level differences but across cities within a country. These findings are consistent with a recent study in India which found that the positive association between diabetes and individual SES in less developed districts became weaker or inverse in districts with higher levels of development.^26^ Our results highlight the importance of considering the different social and economic environments within a region and how the effects of individual-level factors may be modified by environmental conditions.

An important limitation of our study is the use of self-report survey data for the ascertainment of diabetes status. This may have led to underestimates in groups with less access to healthcare and may have consequently resulted in underestimates of educational gradients. Gender differences in access to healthcare, such as prenatal care identification of high-risk women, and pattering of healthcare access by SES could also have impacted the gender differences that we observed. The year of the surveys and census measures are not fully aligned across countries, although some stability in the census-based measures of population characteristics can be assumed.^3^ Because our focus was on estimating associations rather that prevalences for specific cities we did not use survey weights; however, variables related to the weights were included as adjustment factors. Our results are cross sectional and cannot be used to draw causal inferences but still provide valuable descriptive information relevant to policy. Major strengths of our study include the large sample of individuals (110 498) and cities (232) representing a significant proportion of the urban population of Latin America, the harmonised dataset, and the availability of city-specific information absent from past work. Our models are adjusted for country fixed effects that would account for any unmeasured country factors such as differences in healthcare and education systems across countries.

In summary, we found strong educational gradients in diabetes in women living in Latin American cities, regardless of city socioeconomic development. Findings in men suggest that inverse gradients may emerge as country and city socioeconomic development increase. Our results show that social gradients varied and modified by country and local contexts. Considering the increasing trends of diabetes and the high urbanisation in Latin America, it is paramount to develop policies aimed at reducing social inequities in diabetes in cities. These policies need to be sensitive to the ways in which inequities in diabetes manifest in different social and economic contexts and how they may vary across countries and cities. Targeting socioeconomic and gender inequities in cities will be critical to achieving the sustainable development goals related to reducing NCDs in LMIC.

## Supplementary Material

Online supplemental table 1

## Figures and Tables

**Table 1:** City-level and individual-level characteristics by city-level SEI tertile

	Overall	Low SEI	Medium SEI	High SEI	P value
City characteristics					
Number of cities	232	98	64	70	
City-SEI	0.28 (–0.14, 0.46)	–0.36 (–0.76, –0.14)	0.28 (0.18, 0.39)	0.58 (0.46, 0.76)	<0.001
SEI components					
% households with piped water	89.9 (81.2, 95.0)	77.8 (74.8, 87.0)	90.8 (85.6, 95.3)	94.2 (90.3, 95.5)	<0.001
% households with sewage network	78.3 (54.7, 88.0)	52.7 (29.5, 78.3)	75.6 (56.7, 88.0)	87.2 (80.1, 94.4)	<0.001
% households with overcrowding	3.4 (2.1, 6.0)	6.8 (3.7, 10.5)	3.9 (2.4, 5.3)	2.7 (1.7, 3.1)	<0.001
% population 25 and older with at least primary education	79 (72.9, 84.3)	75.2 (69.7, 82.3)	78.3 (72.7, 81.6)	82.9 (76.3, 87.3)	<0.001
Individual-level characteristics					
Number of survey respondents	110 498	37 213	34 939	38 346	
Age	43 (34, 56)	42 (33, 55)	44 (35, 58)	44 (34, 57)	<0.001
Women %	58.9	59.7	58.0	58.9	<0.001
Diabetes %	8.0	7.0	9.0	8.1	<0.001
Education					
Less than primary %	19.3	22.7	18.5	16.8	<0.001
Primary %	33.2	28.3	34.2	37.1	
Secondary %	32.9	36.6	30.8	31.3	
University %	14.6	12.5	16.6	14.8	
Survey respondents per country					
Argentina %	16.7	4.0	34.2	13.2	<0.001
Brazil %	27.5	31.3	29.7	22.0	
Chile %	2.2	0	0.1	6.2	
Colombia %	13.8	8.8	12.6	19.7	
Mexico %	21.2	18.5	22.8	22.3	
Panama %	8.4	7.6	0	69.3	
Peru %	9.1	26.2	0.8	0	
El Salvador %	1.2	3.6	0	0	

We present median, 25th and 75th percentiles unless specified otherwise. P values were estimated using Kruskal-Wallis test for continuous variables and χ_2_ test for discrete variables. City-level SEI: Standardised sum of % of households with piped water, % of households with sewage network, %of household with overcrowding (reversed), and % of population 25 and older with at least primary education.

**Table 2:** ORs of diabetes associated with individual-level education and city-SEI by sex

	Model 1	Model 2†	Model 3‡
Individual and city characteristics*	OR (95% CI)	OR (95% CI)	OR (95% CI)
**Women (n=51 903)**			
City-SEI	1.00 (0.95 to 1.06)	0.96 (0.91 to 1.02)	–
Education level	**0.78 (0.73 to 0.83)**	**0.80 (0.75 to 0.85)**	–
*Random effects*			
Intercept variance (SE)	**0.0798 (0.0197)**	**0.0364 (0.0135)**	–
Education slope variance (SE)	**0.0262 (0.0155)**	**0.0259 (0.0142)**	–
**Men (n=37 246)**			
City-SEI	1.02 (0.96 to 1.09)	0.97 (0.92 to 1.03)	0.98 (0.92 to 1.05)
Education level	0.93 (0.87 to 1.00)	0.95 (0.89 to 1.02)	
Argentina, Brazil, Chile, Colombia, Mexico	–	–	**0.92 (0.86 to 0.99)**
Peru, Panama, El Salvador	–	–	**1.24 (1.04 to 1.49)**
*Random effects*			
Intercept variance (SE)	**0.1004 (0.0240)**	**0.0201 (0.0117)**	**0.0210 (0.0117)**
Education slope variance (SD)	**0.0346 (0.0168)**	**0.0209 (0.0127)**	0.0163 (0.0124)

Bold values have a p value<0.05.

* All models were adjusted for individual age. Education is modelled as a continuous variable, a one-unit increase reflects one higher level of education, for example, less than primary to primary complete. City Social Environment Index (SEI) was standardised to a mean of 0 and an SD of 1. The OR is estimated for a 1 SD (0.58703) difference in SEI.

† Model 2 is adjusted for country fixed effects.

‡ Model 3 includes an interaction between education and different country groups only for men (p for interaction = 0.002). Combinations of the main effect of education and the interaction coefficient were used to derive estimates for different countries.

**Table 3:** ORs (95% CI) of diabetes associated with education for cities with different levels of the Social Environment Index (SEI) and residual education slope variance across cities

		Men*	
	Women	Argentina, Brazil, Chile, Colombia, Mexico	Peru, Panama, El Salvador
	OR (95% CI)	OR (95% CI)	OR (95% CI)
Per unit higher education at the 10th pct of SEI	0.81 (0.73 to 0.90)	1.03 (0.92 to 1.16)	1.32 (1.10 to 1.59)
Per unit higher education at the median SEI	0.80 (0.75 to 0.85)	0.91 (0.85 to 0.97)	1.16 (0.96 to 1.40)
Per unit higher education at the 90th pct of SEI	0.80 (0.73 to 0.87)	0.85 (0.78 to 0.94)	1.09 (0.89 to 1.35)
Variance of education slope	**0.0245 (0.0140)**	0.0146 (0.0120)	
P value for education and SEI interaction	0.834	**0.019**	
P value for education and country groups interaction	–	**0.014**	

Models were adjusted for age, country fixed effects, and education and SEI main effects.

* The model for men also includes the interaction between individual education and country.

## Data Availability

Data are available upon reasonable request. The SALURBAL project welcomes queries from anyone interested in learning more about its dataset and potential access to data. To learn more about SALURBAL’s dataset, visit https://drexel.edu/lac/ or contact the project at salurbal@drexel.edu.

## What is already known on this subject

► Previous evidence from high-income countries reports an inverse association of diabetes with individual-level socioeconomic status; however, studies of the social patterning of diabetes in low-income and middle-income countries (LMIC) are scant. Some evidence suggests that social gradients in diabetes are larger in LMIC that are more urbanised compared with less urbanised ones and that social gradients also vary within regions of LMICs, with larger social gradients observed in more compared with less urbanised areas. However, no studies have examined inequities in a systematic fashion across the growing cities of LMIC or how these gradients may vary across countries or be modified by city characteristics.

## What this study adds

► This is the first study to comprehensively examine social gradients in diabetes across the cities of Latin America. We found that in women there was a clear educational gradient in diabetes: women with higher education levels had a lower proportion of diabetes, with a dose–response pattern for all countries. Furthermore, this association was not modified by city social environment. In men, the association of education with diabetes varied across countries and this association was modified by city social environment as such that an inverse association emerged (or a positive association disappeared) as the city socioeconomic conditions improved. It is paramount to develop policies aimed at reducing social inequities in diabetes which can be expected to increase over time as cities continue to grow and develop unequally.
