# Supplementary material for: Association of education level with diabetes prevalence in Latin American cities and its modification by city social environment
Source: J Epidemiol Community Health. 2021 Feb 4;75(9):874–80. doi: 10.1136/jech-2020-216116 (PMC7611487; doi:10.1136/jech-2020-216116)
Supplement: Supplementary data [file jech-2020-216116supp001.pdf]

Supplementary table 1: Characteristics of the national surveys included in our sample.

Country	Survey	Sample Characteristics	Sampling Strategy	Representation
Argentina	Country: Argentina<br>Survey: Encuesta Nacional de Factores de Riesgo, ENFR (National Risk Factors Survey)	Age: >18 years<br>Total N: 32,365<br>N in SALURBAL: 21,451<br>N ≥ 25[a]: 18,479<br>Year: 2013	Multistage [Aglomerado censal; área (groups of radio censales); household; person 18 years or older]<br>Stratified [population size; education level of head of household]	National, four *localidad* groups based on size, 6 regions, 23 provinces, Ciudad Autonoma de Buenos Aires, and 8 metropolitan areas >500,000 population.
Brazil	Country: Brasil<br>Survey: Pesquisa Nacional de Saúde, PNS (National Health Survey)	Age: All ages<br>Total N: 64,308 adults 18+ years<br>N in SALURBAL: 29,353 adults 18+ years<br>N ≥ 25[a]: 30,425<br>Year: 2013	Multistage [census tracts or groups of census tracts; households; person 18 years or older]<br>Stratified [capital city, metropolitan region, or integrated economic development region, then rest of municipalities; Urban/rural; total household income]	Regions (5) States or federation units (27), state capitals (27), urban and rural, metropolitan areas and development integrated areas
Chile	Country: Chile<br>Survey: Encuesta Nacional de Salud, ENS (National Health Survey)	Age: ≥15 years<br>Total N: 5,293<br>N in SALURBAL: 2,840<br>N ≥ 25[a]: 2,405<br>Year: 2010	Multistage [Comunas; Segments within comunas; household; person 15 years or older]<br>Stratified [urban/rural with three groups of population sizes]	National, Regions (15), urban/rural
Colombia	Country: Colombia<br>Survey: Encuesta Nacional de Salud, ENS (National Health Survey)	Age: 0 - 69 years<br>Total N: 102,677 (41,281 adults 18-69 years)<br>N in SALURBAL: 43,182 (18,824 adults 18-69 years)<br>N ≥ 25[a]: 15,221<br>Year: 2007	Multistage [Municipalities or combination of municipalities if small; Manzanas; household; person adults 18-69 and all children 17 and under]<br>Stratified [region; urbanization of municipal seats; urban/rural municipal population; unsatisfied basic needs]	Region, department, subregion, urban area of municipal capitals, urban/rural, by poverty level
Mexico	Country: Mexico<br>Survey: Encuesta Nacional de Salud y Nutricion, ENSANUT (National Survey for Health and Nutrition)	Age: all ages<br>Total N: 46,277 adults 18+ years [2012]<br>N in SALURBAL: 26,335 adults 18+ years [2012]<br>(2012) N ≥ 25[a]: 23,389<br>Years: 2012	Multistage [AGEB; Manzana (urban) or pseudo-manzanas withig localidades (rural); Households; 1 person within each of the groups (0-4 years, 5-9 years, 10-19 years, 20 years and older, recent medical service user)]<br>Stratified [socioeconomic status of AGEB at the state level]	National, state, metropolitan areas, urban/rural, high/low SES
Panama	Country: Panama<br>Survey: Encuesta Nacional de Salud y Calidad de Vida ENSCAVI (National Survey of Health and Quality of Life)	Age: >=18 years<br>Total N: 25,748<br>N in SALURBAL: 11,394<br>N ≥ 25[a]: 9,234<br>Years: 2007	Multistage [Census segments; Dwellings; Persons >=18 years]<br>Stratified [Indigenous population in province; Urban/rural]	National, District
Peru	Country: Peru<br>Survey: Encuesta Nacional de Demografia y Salud, ENDES (National Survey of Demographics and Health)	Age: All ages<br>Total N: 122,368 (adults 18+ years N=32158)<br>N in SALURBAL: 12,597 adults 18+ years<br>N ≥ 25[a]: 10,012<br>Year: 2016	Multistage [Conglomerado (set of census blocks – urban) or Empadronamiento (set of households – rural); Households; One person within each of the groups (>15 years, females 15-49 years, children <5 years, children <12 years)]<br>Stratified [Department; Urban/Rural]	National, Urban National, Rural National, Natural Region: Lima Metropolitan area, coast/mountain/jungle
El Salvador	Country: El Salvador<br>Survey: Encuesta Nacional de Enfermedades Cronicas no transmisibles en Poblacion Adulta de El Salvador ENECA (National Survey of Noncommunicable Chronic Diseases in the Adult Population of El Salvador)	Age: ≥20 years<br>Total N: 4,817<br>N in SALURBAL: 1,5496<br>N ≥ 25[a]: 1,333<br>Year: 2014-2015	Two-stage [Segmento censal, groups of dwellings (compacto); all household members 20 years and older]	National, Urban National, Rural

a.- N ≥ 25 = individuals included in the analysis comprised of adults 25 and older with complete data for age, education level, and diabetes diagnosis.

Supplementary table 2: City and individual-level characteristics by diabetes status.

	Overall	Diabetes: Yes	Diabetes: No	P value
**City-level characteristics**				
City-SEI	0.28 (-0.14,0.46)	0.31 (-0.09,0.46)	0.28 (-0.14,0.46)	<0.001
**SEI components**				
% households with piped water	89.93 (81.21,95.02)	90.82 (81.33,95.34)	89.90 (81.21,95.02)	<0.001
% households with sewage network	78.31 (54.73,88.03)	77.83 (54.71,88.01)	78.31 (54.70,88.01)	0.032
% household with overcrowding	3.42 (2.14,6.04)	3.74 (2.22,6.30)	3.44 (2.13,6.02)	0.038
% population 25 and older with at least primary education	79.03 (72.91,84.34)	79.60 (72.91,84.42)	79.01 (72.92,84.30)	<0.001
**Individual-level characteristics**				
Number of survey respondents	110,498	8,841	101,657	<0.001
Age	43 (34,56)	59 (49,68)	42 (33,55)	<0.001
Women %	58.9	60.6	58.7	<0.001
Education				
Less than primary %	19.3	34.2	18.0	<0.001
Primary %	33.2	34.8	33.1	
Secondary %	32.9	21.2	33.9	
University %	14.6	9.8	15.0	
Survey respondents per country				
Argentina %	16.7	20.4	16.4	<0.001
Brazil %	27.5	26.5	27.6	
Chile %	2.2	2.5	2.2	
Colombia %	13.8	7.5	14.3	
Mexico %	21.2	29	20.5	
Panama %	8.4	7.7	8.4	
Peru %	9.1	4.9	9.4	
El Salvador %	1.2	1.6	1.2	

We present median, 25th, and 75th unless specified otherwise. P-values were estimated using Wilcoxon test for continuous variables and chi-square test for discrete variables.

City-level SEI: Standardized sum of %of households with piped water, % of households with sewage network, %of household with overcrowding (reversed), % of households with durable walls, and % of population 25 and older with at least primary education.
